# Clinical outcomes and prognostic factors in pediatric acute myeloid leukemia with monocytic differentiation: a single-center cohort study from Egypt

**DOI:** 10.3389/fonc.2026.1969723

**Published:** 2026-10-12

**Authors:** Tarek Ahmed Abdelrehim Ahmed, Mahmoud Hammad, Sonya Soliman, Nahla Elsharkawy, Sherine Ibrahim Salem, Sonia Ahmed, Alaa Elhaddad

**Affiliations:** 1 Department of Clinical Oncology and Nuclear Medicine, Sohag University, Sohag, Egypt; 2 Department of Pediatric Oncology, National Cancer Institute (NCI), Cairo University, Cairo, Egypt; 3 Department of Pediatric Oncology, Children’s Cancer Hospital, Cairo, Egypt; 4 Department of Clinical Pathology, National Cancer Institute, Cairo University, Cairo, Egypt; 5 Department of Clinical Pathology, Children’s Cancer Hospital, Cairo, Egypt

**Keywords:** acute myeloid leukemia, FAB M4/M5 subtypes, hematopoietic stem cell transplantation, measurable residual disease, monocytic differentiation, prognostic factors, risk stratification

## Abstract

**Background:**

Acute myeloid leukemia (AML) with monocytic differentiation (FAB M4/M5 subtypes) is a distinct subtype of pediatric AML characterized by aggressive clinical behavior and common extramedullary involvement. Despite therapeutic advances, outcomes remain suboptimal, particularly in resource-limited settings. The study aimed to evaluate the clinical characteristics, treatment outcomes, and prognostic markers in pediatric patients with AML, FAB M4/M5 subtypes.

**Patients and methods:**

A retrospective cohort study analyzing 193 patients with AML, FAB M4/M5 subtypes diagnosed at the Children’s Cancer Hospital, Egypt-57357 (CCHE-57357) between January 2016 and December 2021.

**Results:**

The cohort comprised 131 patients with M4 and 62 with M5 morphology. Median follow-up was 62.0 months among survivors and 37.6 months for the entire cohort. Three- and five-year OS were 53.1% and 48.3%; three- and five-year EFS were 48.2% and 45.5%. Five-year CIR was 23.8%. Forty-eight patients (24.9%) died during induction. Multivariate Cox regression identified MRD positivity after first induction (HR 2.868, 95% CI 1.133–7.260, *p* = 0.026) and monosomy 7 (HR 6.206, 95% CI 2.692–14.306, *p* < 0.001) as independent adverse prognostic factors. Conversely, inv(16)(p13.1;q22) or t(16;16)(p13.1;q22); CBFB::MYH11 was an independent favorable prognostic factor (HR 0.253, 95% CI 0.108–0.592, *p* = 0.002). 14 high-risk pediatric AML patients underwent HSCT post-induction; three experienced adverse outcomes, comprising relapses (*n* = 2), and acute GVHD (*n* = 1).

**Conclusion:**

Genetic findings and treatment response were associated with outcome, supporting a complete diagnostic assessment. Improving survival requires precise risk stratification, MRD-guided therapy, and robust supportive care.

## 1 Introduction

Acute myeloid leukemia (AML) is the second most common childhood hematological malignancy, accounting for 15-20% of pediatric leukemias (1). Despite major advances in survival, EFS rates have improved only modestly, from 41% to approximately 50–65% (1, 2). Furthermore, 24–41% still experience relapse (3, 4).

Pediatric AML with monocytic differentiation (FAB M4/M5) is an aggressive subtype, accounting for 25-30% of cases (5, 6). It has been associated with a higher incidence of hyperleukocytosis and extramedullary infiltration, as well as lower survival compared with other AML subtypes (7–9).

The prognostic significance of specific chromosomal abnormalities in AML has been well-established since 1984, guiding risk-adapted therapeutic strategies (10–12). inv(16)(p13.1;q22) or t(16;16)(p13.1;q22); CBFB::MYH11 has been recognized as a favorable prognostic marker, especially in AML-M4 (12, 13). On the other hand, FAB-M5 has been linked to higher cytogenetic risk, and lower survival in some studies (10, 14, 15). However, some reports suggest that remission rates and OS in AML-M5 may not differ significantly from other AML subtypes, and that outcomes are highly influenced by genetic alterations and molecular risk stratification (16, 17).

Another challenge is that survival and outcomes in resource-limited settings frequently fall below those reported in high-income countries, making the role of genetic abnormalities in AML classification, management, and prognosis prediction even more important, especially in such settings, where patients may face distinct diagnostic and therapeutic challenges (17–20).

The objective of this study was to present a comprehensive analysis of 193 pediatric patients with AML with monocytic differentiation (FAB M4 and M5) treated at a single center in Egypt according to the Children’s Cancer Hospital, Egypt-57357 (CCHE 57357) AML protocol, adapted from the Children’s Oncology Group AAML1031 protocol, over a six-year period. This represents one of the largest reported series focusing exclusively on monocytic AML in a resource-limited setting.

## 2 Patients and methods

### 2.1 Study design and patient characteristics

This retrospective cohort study was conducted at Children’s Cancer Hospital Egypt-57357. From January 2016 through December 2021, 200 children and adolescents with FAB M4 or M5 AML were screened. Five patients with Fanconi anemia treated with an alternative FLAG-based induction regimen, one patient with secondary AML evolving from myelodysplastic syndrome, and one patient who died before treatment initiation were excluded from the treatment-initiated analysis, leaving 193 patients (**Figure 1**).

**Figure 1:**
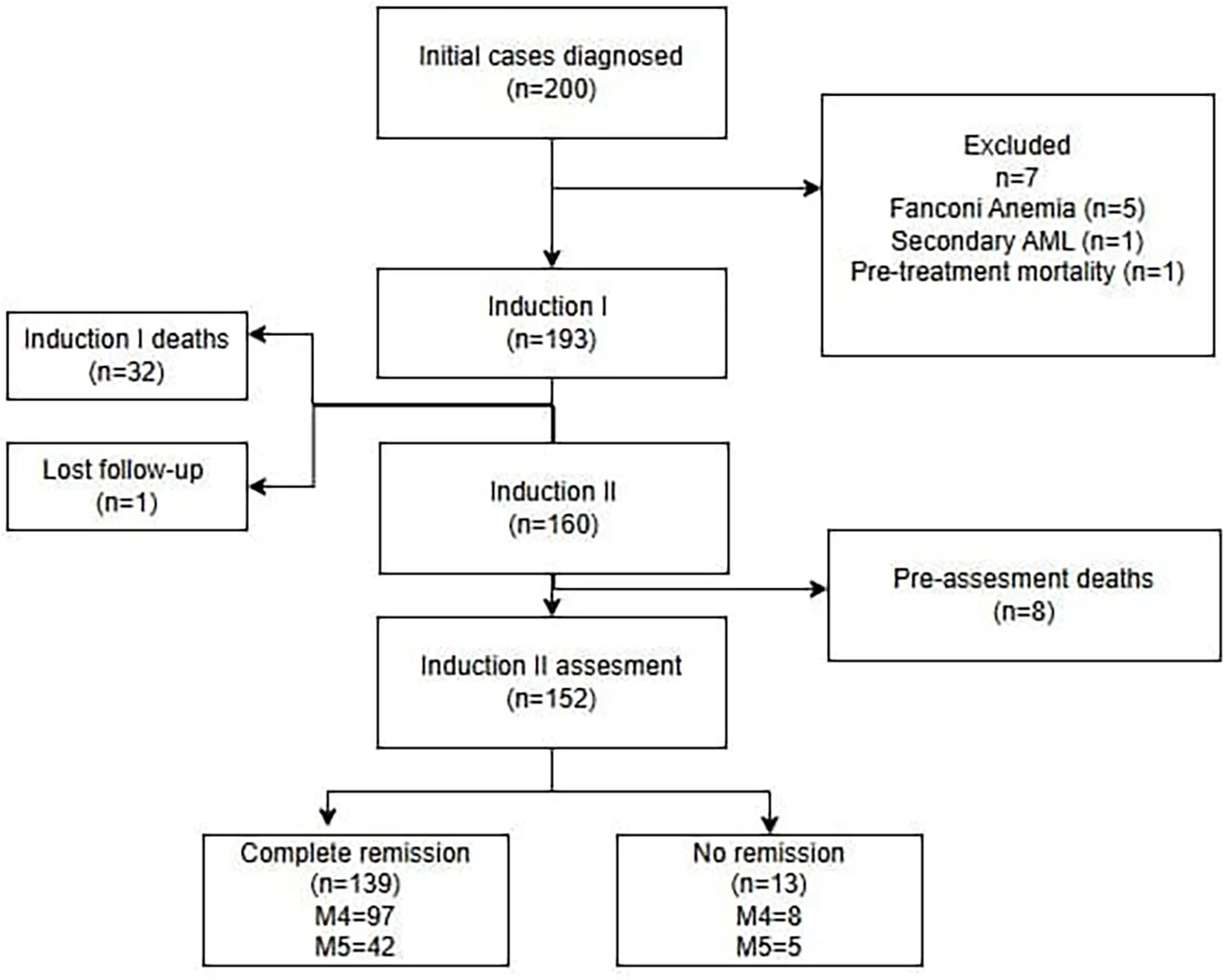
Flow diagram of the study cohort. Flowchart illustrating patient selection, eligibility criteria, induction treatment progression, and final assessment cohorts at Children’s Cancer Hospital Egypt (CCHE-57357).

Additional exclusion criteria were acute promyelocytic leukemia, therapy-related or relapsed AML, isolated extramedullary myeloid sarcoma, myelodysplastic syndrome, and Down syndrome–associated AML.

### 2.2 Clinical and laboratory assessment at diagnosis

Baseline clinical assessment included a detailed history, physical examination, and performance status evaluation. Laboratory investigations comprised complete blood count, liver and renal function tests, and tumor lysis syndrome (TLS) monitoring using the Cairo-Bishop criteria (21). Bone marrow aspirates were evaluated for morphology, cytochemical staining, immunophenotyping, cytogenetics, and molecular testing.

### 2.3 Diagnostic criteria and classification

FAB classification used Wright–Giemsa–stained peripheral blood and marrow smears. M4 required granulocytic and monocytic differentiation with at least 20% of nonerythroid cells showing monocytic features; M5 required at least 80% monocytic-lineage differentiation (22).

### 2.4 Immunophenotyping analysis

MRD assessment was done for all patients after each cycle of treatment by flow cytometry. Flow cytometry was conducted using the Navios EX instrument, equipped with ten colors and three lasers. Typically, approximately one million cells are acquired; however, in instances of hypocellular bone marrow (BM), the cell count is lower. The gating strategy involved two tubes: one for the “Leukemia-associated immunophenotype” (LAIP) and another designated for “different-from-normal” analysis. Both approaches were employed concurrently. The positivity threshold, with a sensitivity/limit of detection (LOD) of less than 0.1%, was established. For quality control, daily flow set and flow check procedures were implemented. Additionally, cases of BM without infiltration, such as those from lymphoma, solid tumors, and donors of BM transplantation, were utilized prior to initiating minimal residual disease (MRD) detection.

⁠Limit of Detection (LOD) studies: assess the minimum detectable level of aberrant cells. Limit of Quantification (LOQ) studies: evaluate the lowest level of MRD that can be accurately quantified. ⁠Linearity studies: assess the assay’s ability to detect MRD across a range of concentrations. ⁠Precision studies: evaluate intra- and inter-assay variability. ⁠Comparison with other methods: correlate flow cytometry results with molecular or other techniques (e.g., PCR, NGS) (23, 24).

### 2.5 Molecular and cytogenetic analysis

Cytogenetic analysis of the bone marrow aspirate samples was performed using conventional karyotyping in accordance with ISCN 2020 guidelines, supplemented by fluorescence *in situ* hybridization (FISH). Conventional karyotyping was used to identify numerical and structural chromosomal abnormalities, including complex karyotype (defined as ≥3 chromosomal abnormalities), monosomy 7, and monosomy 5. FISH was performed to detect the following recurrent cytogenetic abnormalities: inv (16)(p13.1;q22) or t (16;16)(p13.1; q22); CBFB::MYH11, t (8;21)(q22;q22.1); RUNX1::RUNX1T1, and KMT2A (MLL) rearrangements at chromosome 11q23.

Molecular testing of the bone marrow aspirate samples was performed using polymerase chain reaction (PCR). Markers assessed included FLT3-ITD (FMS-like tyrosine kinase 3 internal tandem duplication), with a high allelic ratio defined as ≥ 0.4, NPM1 (nucleophosmin 1 mutation); CEBPA (CCAAT/enhancer-binding protein alpha mutation); WT1 (Wilms tumor 1 overexpression); and TP53 mutation. Not all molecular and cytogenetic markers were assessed in every patient, primarily due to sample availability. The number of patients tested for each marker is specified according to the results.

### 2.6 Risk stratification and treatment

Patients were stratified based on cytogenetics and molecular abnormalities into: Low risk (LR) group including patients with favorable cytogenetics, including t(8;21) (q22;q22.1); RUNX1::RUNX1T1, inv (16) (p13.1;q22) or t (16;16) (p13.1;q22); CBFB::MYH11), nucleophosmin 1 (NPM1), and CEBPA mutations, while High risk (HR) included those with unfavorable cytogenetics, such as monosomy 7, monosomy 5, complex karyotype (more than three chromosomal aberrations), and FLT3 internal tandem duplications (FLT3/ITD) with a high allelic ratio (≥ 0.4) and Intermediate risk (IR) included patients lacking both favorable and unfavorable markers.

Following the first induction, IR patients were reclassified into HR and LR categories based on measurable residual disease (MRD) assessed by multiparametric flow cytometry (MFC). LR patients were defined as those with MRD <0.1%, while HR patients were those with MRD ≥ 0.1%. The 0.1% MRD threshold was selected based on established convention in pediatric AML flow cytometry-based MRD assessment, consistent with prior cooperative group studies and consensus guidelines (Children’s Oncology Group AAML0531 trial by Gamis et al., (25); European LeukemiaNet MRD consensus by Schuurhuis et al., (24); St. Jude AML02 trial by Rubnitz et al., (26)) (24–26).

All patients were treated with the CCHE-57357 AML protocol adapted from the COG AAML1031 protocol (clinicaltrials.gov NCT01371981) throughout the entire study period (25, 27). The treatment protocol consisted of four planned chemotherapy cycles. High-risk patients in first complete remission (CR) were offered allogeneic hematopoietic stem cell transplantation (Allo-HSCT) if they had a matched sibling donor (detailed treatment is shown in the supplement) (**Supplementary Figure 1**). Standard antimicrobial prophylaxis consisting of levofloxacin, voriconazole, and trimethoprim-sulfamethoxazole and empiric management of infectious complications were administered consistently across the cohort in accordance with institutional guidelines.

### 2.7 Response definitions and outcome measures

Complete remission (CR): defined as < 5% blasts by morphology and no extramedullary disease. Refractory disease was defined as ≥5% BM blasts, morphologically or by proven extramedullary disease, at the end of induction therapy. Relapses were defined as the reappearance of blasts in peripheral blood (PB) or bone marrow (BM) or the development of extramedullary disease after initial morphologic CR.

Mortality outcomes were categorized by timing and etiology. Early mortality was defined as death from any cause within 14 days of treatment initiation. Disease-related mortality (DRM) was defined as death driven by primary disease burden (e.g., leukostasis, hemorrhage) or occurring with evidence of active, progressive, or refractory leukemia. Treatment-related mortality (TRM) was defined as death attributable to treatment toxicity or clinical complications (e.g., sepsis) in the absence of progressive or refractory disease.

Event-free survival (EFS) was defined as the time from diagnosis to the earliest event, including relapse, progression, second malignancy, or death from any cause. Overall survival (OS) was measured from diagnosis to death from any cause.

### 2.8 Statistical analysis

Statistical analysis was performed using IBM SPSS Statistics v23. Categorical variables were expressed as frequencies and percentages, and continuous variables as means ± standard deviation (SD) or medians with ranges. Categorical variables were compared using the chi-square or Fisher’s exact test as appropriate, and continuous variables using the Mann-Whitney or Welch two-sample t-test. Overall and event-free survival probabilities (OS, EFS) were calculated using Kaplan-Meier analysis, and comparisons between groups were performed using the log-rank test. Cumulative incidence of relapse (CIR) was defined as the time from achievement of complete remission to relapse, death, or last follow-up, with death without relapse treated as a competing event; CIR was estimated using the cumulative incidence function and compared using Gray’s test using R software (version 4.6.0). The impact of risk factors on OS and EFS was assessed through univariable Cox regression. Variables for the multivariable Cox proportional hazards model were selected based on clinical relevance and univariable Cox Regression with outcome (P < 0.1), with individual cytogenetic markers with fewer than 5 events excluded given the risk of unstable estimates. The final model included six covariates (age, inv(16), complex karyotype, monosomy 7, hyperleukocytosis, and MRD status after induction I), evaluated against 104 events, consistent with a minimum of 10 events per covariate. Multicollinearity was assessed, and no substantial collinearity was identified among the included covariates (variance inflation factors <1.1 for all variables). Due to variations in sample availability, cytogenetic and molecular investigations were not uniformly completed for all patients. Consequently, frequencies for each specific genetic marker or abnormality are reported strictly based on the number of successfully evaluated patients, with variable denominators specified for each parameter. All p-values were two-sided, with p < 0.05 considered significant.

## 3 Results

In this cohort of 193 pediatric patients diagnosed with acute myeloid leukemia with monocytic differentiation, 131 (67.9%) presented with M4 morphology, while 62 (32.1%) exhibited M5 morphology. The median age was 7.5 years, with a range spanning from 0.3 to 17.9 years. Among the patients, 12 (6.2%) were infants, 114 (59.1%) were aged between 1 and less than 10 years, and 67 (34.7%) were aged 10 years or older. Notably, age distribution varied significantly by FAB subtype (*p* = 0.001): M5 was more prevalent among infants (14.5% *vs*. 2.3%), whereas M4 was more common among those aged 10 years or older (39.7% *vs*. 24.2%) (*p* = 0.001). The cohort included 103 males (53.4%) and 90 females (46.6%), yielding a male-to-female ratio of 1.14:1, with no significant differences observed between subtypes.

The median presenting white-cell count was 31.3 × 10^9^/L, ranging from 1.0 to 417.8 × 10^9^/L. Hyperleukocytosis was identified in 42 patients (21.8%): comprising 31 of 131 (23.7%) with M4 and 11 of 62 (17.7%) with M5 (*p* = 0.018).

Hepatosplenomegaly (80.3%) and fever (64.8%) emerged as the most common presenting clinical features in the cohort. Shortness of breath was noted in 17 patients (8.8%), altered consciousness in 10 (5.2%), gingival hyperplasia in 9 (4.7%), and myeloid sarcoma in another 9 patients (4.7%). The myeloid sarcoma was localized to the orbit in 3 patients, to intracranial sites in 3, and to the cervical, buccal, and temporalis regions in 1 patient each. Additionally, renal parenchymal infiltration was incidentally identified via abdominal imaging in 4 patients (2.1%). No significant differences in presenting clinical features were observed between the subtypes. The patients’ clinical characteristics are summarized in (**Table 1**).

**Table 1:** Clinical characteristics of pediatric acute myeloid leukemia with monocytic differentiation, by FAB subtype.

Characteristic	Whole cohort (%) N=193	M4 subtype (%) N=131	M5 subtype (%) N=62	*p*-value
Demographics				
Age, years				
<1	12 (6.2)	3 (2.3)	9 (14.5)	0.001*
1 to <10	114 (59.1)	76 (58.0)	38 (61.3)	
≥10	67 (34.7)	52 (39.7)	15 (24.2)	
Sex				
Male	103 (53.4)	74 (56.5)	29 (46.8)	0.207
Female	90 (46.6)	57 (43.5)	33 (53.2)	
WBC count, × 10^9^/L				
<50	121 (62.7)	74 (56.5)	47 (75.8)	0.018*
50–99.9	30 (15.5)	26 (19.8)	4 (6.5)	
≥100	42 (21.8)	31 (23.7)	11 (17.7)	
Presenting features				
Hepatosplenomegaly	155 (80.3)	108 (82.4)	47 (75.8)	0.279
Fever	125 (64.8)	79 (60.3)	46 (74.2)	0.074
Shortness of breath	17 (8.8)	10 (7.6)	7 (11.3)	0.519
Altered consciousness	10 (5.2)	5 (3.8)	5 (8.1)	0.296
Gingival hyperplasia	9 (4.7)	5 (3.8)	4 (6.5)	0.472
Myeloid sarcoma (chloroma)	9 (4.7)	4 (3.1)	5 (8.1)	0.149
Renal infiltration	4 (2.1)	1 (0.8)	3 (4.8)	0.098
Cytogenetic and molecular findings	Whole cohort N=193 n/tested(%)	M4 subtype N=131 n/tested(%)	M5 subtype N=62 n/tested (%)	*p*-value
Favorable				
inv(16)(p13.1;q22) or t(16;16)(p13.1;q22); CBFB::MYH11	50/190 (26.3)	44/129 (34.1)	6/61 (9.8)	<0.001*
NPM1 mutation	7/120 (5.8)	7/84 (8.3)	0/36 (0.0)	0.101
t(8;21) (q22;q22.1); RUNX1::RUNX1T1	13/190 (6.8)	13/128 (10.2)	0/62 (0.0)	0.006*
CEBPA mutation	3/84 (3.6)	2/61 (3.3)	1/23 (4.3)	NE
Intermediate				
Normal karyotype	43/190 (22.6)	34/129 (26.4)	9/61 (14.8)	0.074
KMT2A rearrangement	49/190 (25.8)	14/129 (10.9)	35/61 (57.4)	<0.001*
Unfavorable				
Monosomy 7	8/190 (4.2)	7/129 (5.4)	1/61 (1.6)	0.440
Monosomy 5	1/190 (0.5)	0/129 (0.0)	1/61 (1.6)	NE
Complex karyotype	5/190 (2.6)	1/129 (0.8)	4/61 (6.6)	NE
FLT3-ITD (allelic ratio ≥0.4)	8/180 (4.4)	7/124 (5.6)	1/56 (1.8)	0.440
TP53 mutation	3/144 (2.1)	2/96 (2.1)	1/48 (2.1)	NE
WT1 overexpression				
Yes	72/93 (77.4)	56/65 (86.2)	16/28 (57.1)	0.002*
No	21/93 (22.6)	9/65 (13.8)	12/28 (42.9)	
Risk stratification				
Initial risk				
Low	71 (36.8)	64 (48.9)	7 (11.3)	<0.001*
Intermediate	101 (52.3)	53 (40.5)	48 (77.4)	
High	21 (10.9)	14 (10.7)	7 (11.3)	
At end of induction I (EOI I)				
Low	65 (33.7)	56 (42.7)	9 (14.5)	<0.001*
High	99 (51.3)	59 (45.0)	40 (64.5)	
Not available	29 (15.0)	16 (12.2)	13 (21.0)	
Five-year outcomes				
Overall survival (OS), %	48.3	51.9	40.6	0.086
Event-free survival (EFS), %	45.5	48.2	40.1	0.195
Cumulative incidence of relapse (CIR), %	23.8 (95% CI, 17.7–30.2)	24.2	22.8	0.859

Values are n (%) unless otherwise indicated. Molecular and cytogenetic frequencies are reported among patients with available testing. P values were calculated using the *χ*^2^ test or Fisher’s exact test, as appropriate. NE, not estimable, due to small number of cases.

WBC, white blood cell count; FAB, French-American-British; NPM1, nucleophosmin 1; CEBPA, CCAAT enhancer-binding protein alpha; FLT3-ITD, FMS-like tyrosine kinase 3 internal tandem duplication; EOI, end of induction. *significant p value <0.05.

### 3.1 Cytogenetic and molecular characteristics

Among the patients assessed, inv(16)(p13.1;q22) or t(16;16)(p13.1;q22); CBFB::MYH11 alteration was identified in 50 of 190 patients (26.3%), with significantly higher prevalence in the M4 subtype compared with M5 (34.1% *vs*. 9.8%; *p* < 0.001). The t(8;21) (q22;q22.1); RUNX1::RUNX1T1 fusion was exclusively observed in 13 of 190 patients (6.8%), all belonging to the M4 subtype (*p* = 0.006).

KMT2A rearrangements were present in 49 of 190 patients (25.8%) and were significantly more common in the M5 subtype than M4 (57.4% *vs*. 10.9%; *p* < 0.001). Additionally, these rearrangements were more frequently detected in infants compared to older children (63.6% *vs*. 23.5%; *p* = 0.003).

Other genetic alterations included FLT3 internal tandem duplication in 8 of 180 patients (4.4%). NPM1 mutations in 7 of 120 (5.8%), CEBPA mutations in 3 of 84 (3.6%), complex karyotypes in 5 of 190 (2.6%), monosomy 7 in 8 of 190 (4.2%), monosomy 5 in 1 of 190, TP53 in 3 of 144 (2.1%), and WT1 overexpression in 72 of 93 (77.4%) which was observed more frequently in the M4 subtype (*p* = 0.002).

Initial risk stratification varied significantly by subtype (*p* < 0.001). M4 subtype exhibited a predominance of favorable risk, with rates of 48.9% compared to 11.3% in the M5 subtype. In contrast, M5 showed a higher prevalence of intermediate risk at 77.4%, as opposed to 40.5% in M4 (*p* < 0.001). Similar trends were observed in end-of-induction 1 risk classification: low risk was more prevalent in M4 (42.7% *vs*. 14.5%), while high risk was more common in M5 (64.5% *vs*. 45.0%) (*p* < 0.001) (**Table 1**).

### 3.2 Response to induction therapy

Of the 193 patients who received induction I, 164 (85.0%) had a documented morphologic and MRD assessment, 29 died prior to assessment, while 3 achieved complete morphologic remission (M1) on assessment but died shortly thereafter before a second induction course could be initiated, resulting in 32 patients who died before proceeding to induction II.

Following induction I, 94 of 164 evaluable patients (57.3%) attained a marrow blast < 5% with no significant difference between FAB subtype (*p* = 0.708); 138 of 164 (84.1%) remained MRD-positive (*p* = 0.914). After induction II, 139 of 152 evaluable patients (91.4%) had < 5% blasts (*p* = 0.540), and 62 of 152 (40.8%) remained MRD-positive (*p* = 0.312) (**Table 2**).

**Table 2:** Treatment response after induction I and induction II.

Response or assessment	Whole cohort n/N (%)	M4 subtype n/N (%)	M5 subtype n/N (%)	*p*-value
Morphologic response after induction I				
**Patients assessed, n**	164	115	49	0.708
<5% blasts	94/164 (57.3)	67/115 (58.3)	27/49 (55.1)	
≥5% blasts	70/164 (42.7)	48/115 (41.7)	22/49 (44.9)	
Not assessed	29/193 (15.0)	16/131 (12.2)	13/62 (21.0)	
MRD by flow cytometry after induction I				
<0.1%	26/164 (15.9)	18/115 (15.7)	8/49 (16.3)	0.914
≥0.1%	138/164 (84.1)	97/115 (84.3)	41/49 (83.7)	
Not assessed	29/193 (15.0)	16/131 (12.2)	13/62 (21.0)	
Morphologic response after induction II				
**Patients assessed, n**	152	105	47	0.540
<5% blasts	139/152 (91.4)	97/105 (92.4)	42/47 (89.4)	
≥5% blasts	13/152 (8.6)	8/105 (7.6)	5/47 (10.6)	
Not assessed	12/164 (7.3)	10/115 (8.7)	2/49 (4.1)	
MRD by flow cytometry after induction II				
<0.1%	90/152 (59.2)	65/105 (61.9)	25/47 (53.2)	0.312
≥0.1%	62/152 (40.8)	40/105 (38.1)	22/47 (46.8)	
Not assessed	12/164 (7.3)	10/115 (8.7)	2/49 (4.1)	

Values are n/N (%) unless otherwise indicated. P values were calculated using the chi-square test or Fisher's exact test, as appropriate. MRD, measurable residual disease; n, number.

### 3.3 Hematopoietic stem cell transplantation

Among the 14 patients with high-risk stratification who received HSCT in first complete remission (CR1), 11 patients continued to have sustained complete remission while 3 experienced adverse outcomes. These included disease relapses in 2 patients and acute graft-versus-host disease (GVHD) in 1 patient.

### 3.4 Survival analysis

With a median follow-up of 62.0 months for survivors (37.6 months for the whole cohort), 3- and 5-year OS were 53.1% and 48.3%, and 3- and 5-year EFS were 48.2% and 45.5%. The 5-year CIR for the whole cohort was 23.8% (95% CI, 17.7 to 30.2) (**Figures 2A–C**). Infants exhibited significantly poorer 5-year EFS 25.0% (95% CI: 9.4–67.0, *p* = 0.028) and OS 25% (95% CI: 9.0–67.0, *p* = 0.012) when compared with older children. The CIR 16.7%, however, did not show any significant difference (95% CI: 0.0–40.3, *p* = 0.321) (**Figures 3A–C**). Notably, infants had the highest rate of non-relapse early mortalities, (7/12, 58.3%), compared with 26.3% (30/114) in the 1–9-year group and 34.3% (23/67) in the ≥10-year group (Gray’s test for non-relapse mortality: *p* = 0.014).

**Figure 2:**
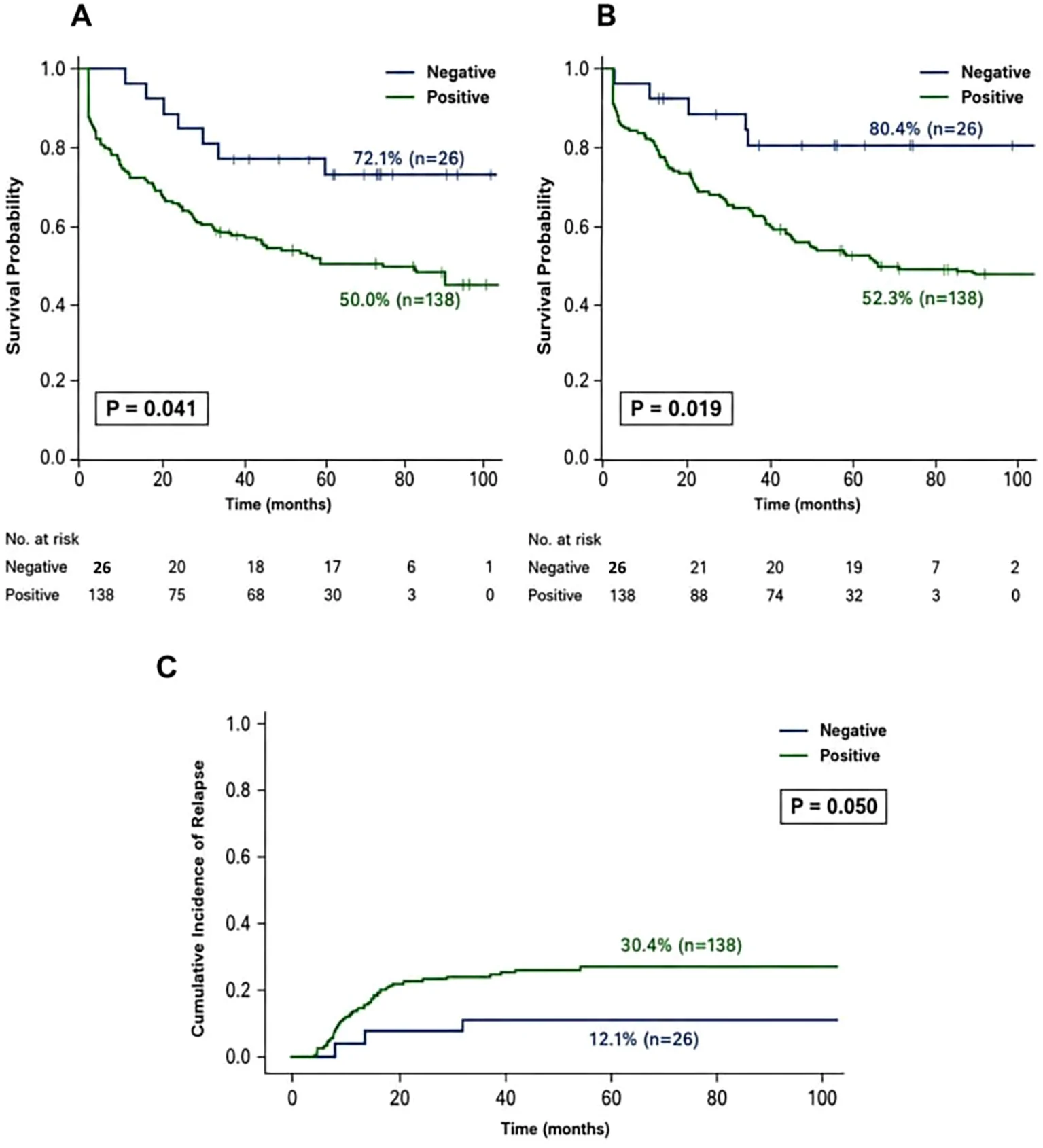
Overall survival, event-free survival, and relapse incidence for the overall cohort. Kaplan–Meier survival curves showing **(A)** 5-year event-free survival (EFS, 45.5%), **(B)** 5-year overall survival (OS, 48.3%), and **(C)** 5-year cumulative incidence of relapse (CIR, 23.8%) for the total cohort ( n=193). Vertical tick marks represent censored observations. Numbers at risk at indicated time points are displayed below the *x*-axes in panels **(A, B)**.

**Figure 3:**
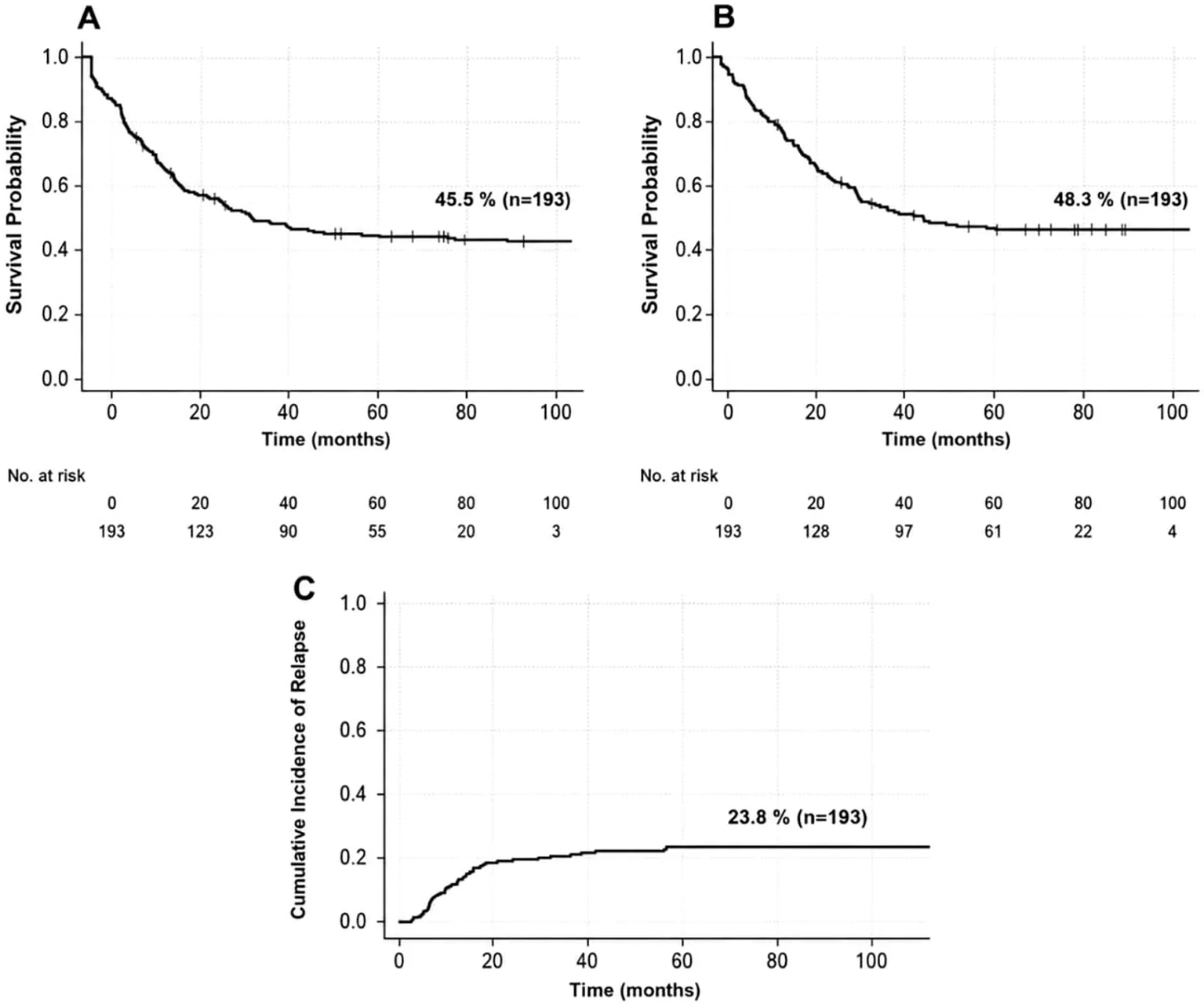
Survival outcomes stratified by age at diagnosis. Kaplan–Meier curves and cumulative incidence of relapse plots comparing infants (< 1 year, n=12) versus non-infant children (≥ 1 year, n=181): **(A)** Event-free survival, **(B)** overall survival, and **(C)** cumulative incidence of relapse. Log-rank *p*-values are shown for survival outcomes (panels **(A, B)**), and Gray’s test *p*-value is shown for CIR (panel **(C)**).

Males had slightly better 5-year EFS and OS (50.2% and 50.6%, respectively) than females (40% and 45.6%, respectively), although these differences were not statistically significant. The M4 FAB subtype showed higher 5-year EFS and OS (48.2% and 51.9%, respectively) than M5 (5-year EFS: 40.1%, 5-year OS: 40.6%), although this difference was not statistically significant (*p* = 0.195, 0.086 respectively). The WBC count at presentation differed significantly between FAB subtypes (*P* = 0.018). M5 patients were more frequently represented in the <50 × 10^9^/L category (75.8% *vs*. 56.5%), whereas M4 patients were more frequently represented in the 50–99.9 × 10^9^/L category (19.8% *vs*. 6.5%). Patients with WBC <100 × 10^9^/L had better 5-year EFS (48.5%, log rank *p* = 0.037) and 5-year OS (51.7%, log rank *p* = 0.032) compared to hyperleukocytosis (WBC ≥ 100 × 10^9^/L).

Patients with inv(16)(p13.1;q22) or t(16;16)(p13.1;q22); CBFB::MYH11 was associated significantly superior outcomes, with 5-year EFS of 67.9% (95% CI: 56.0-82.0, log rank *p* = 0.004), 5-year OS of 71.6% (95% CI: 60.0-86.0, log rank *p* = 0.002) (**Figures 4A–C**). t(8;21) (q22;q22.1); RUNX1::RUNX1T1 was similarly associated with favorable 5-year EFS (76.9%, log rank *p* = 0.048), although the corresponding 5-year OS and CIR differences did not reach statistical significance (*p* = 0.071 and *p* = 0.186, respectively). NPM1-mutated patients showed numerically favorable but non-significant 5-year EFS and 5-year OS (*p* = 0.225 and *p* = 0.310, respectively). Neither normal karyotype nor KMT2A rearrangement was significantly associated with 5-year EFS, 5-year OS, or CIR. FLT3 ITD was associated with a significantly elevated CIR (62.5%, *p* = 0.012), although this finding should be interpreted cautiously given the small number of positive patients (*n* = 8). Complex karyotyping, monosomy 5, TP53 mutation, and WT1 overexpression were not statistically significantly associated with 5-year EFS, 5-year OS, or CIR.

**Figure 4:**
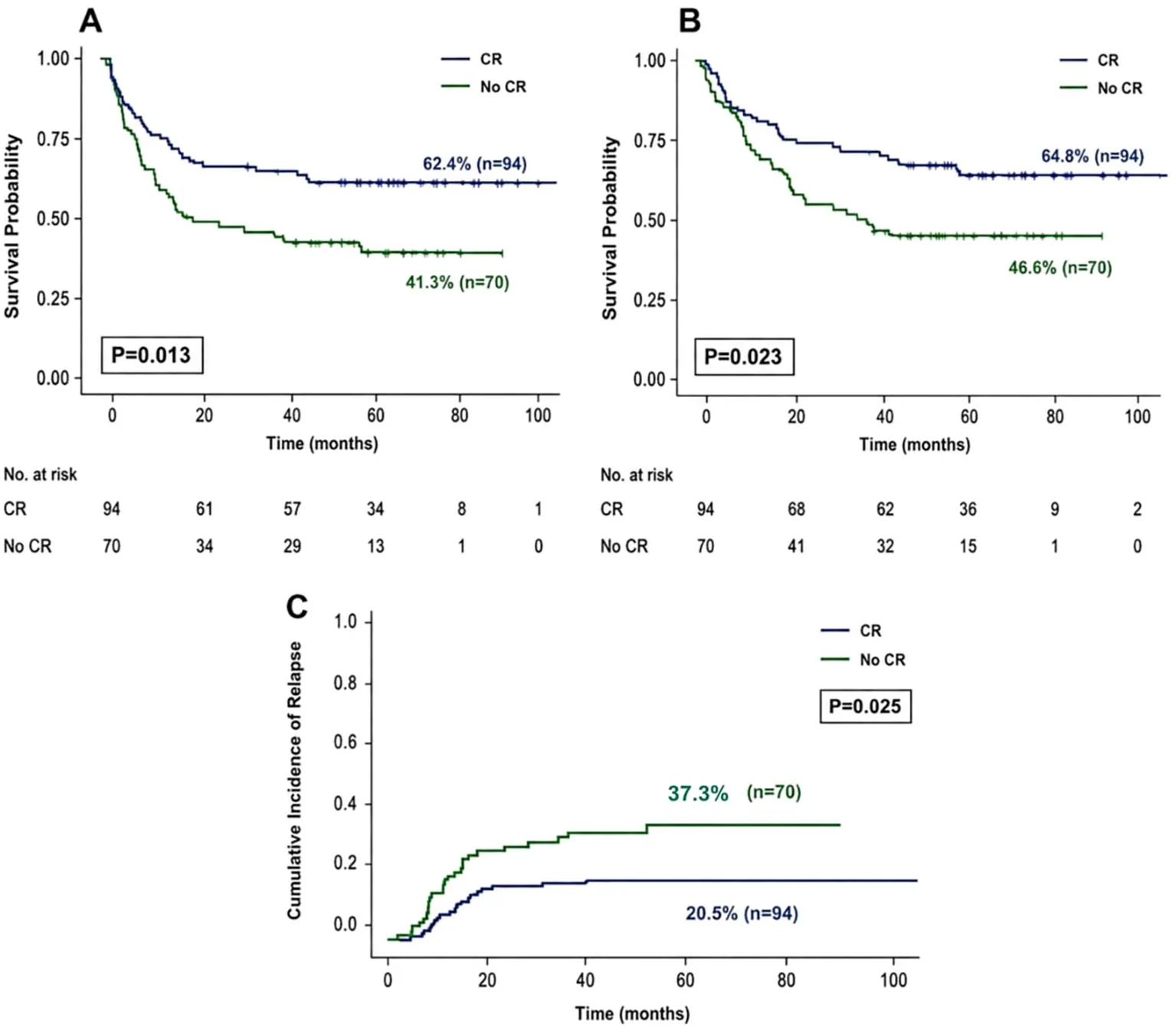
Prognostic impact of inv(16)(p13.1;q22)/CBFB::MYH11. Kaplan–Meier survival curves and cumulative incidence of relapse plots for patients harboring inv(16) ( n=50) compared to inv(16)-negative patients ( n=140): **(A)** 5-year EFS, **(B)** 5-year OS, and **(C)** 5-year CIR. Log-rank *p*-values (panels **(A, B)**) and Gray’s test *p*-value (panel **(C)**) are indicated on each plot.

Of the 8 patients with monosomy 7, 5 died during treatment, 2 had refractory disease, and 1 relapsed, no patients survived beyond 42.8 months, which is the time of the last observed death in this subgroup. The CIR difference was not statistically significant, reflecting non-relapse deaths. These estimates should, however, be interpreted with caution given the small number of this subgrou*p* (*n* = 8).

Morphologic remission after induction I was associated with significantly superior 5-year EFS (62.4% *vs*. 41.3%, log rank *p* = 0.013), 5-year OS (64.8% *vs*. 46.6%, log rank *p* = 0.023), and lower CIR (20.5% *vs*. 37.3%, *p* = 0.025) compared with no CR (**Figures 5A–C**). Similarly, measurable residual disease (MRD) < 0.1% by flow cytometry at the end of induction I (EOI I) was associated with superior 5-year EFS (72.1% *vs*. 50%, log rank *p* = 0.041), 5-year OS (80.4% *vs*. 52.3%, log rank *p* = 0.019) and a trend towards a lower CIR (12.1% *vs*. 30.4%, *p* = 0.050) compared with MRD ≥ 0.1% (**Figures 6A–C**). Risk stratification at EOI I, incorporating cytogenetic and MRD data, was strongly associated with outcome: low-risk patients had 5-year EFS of 70.4% versus 39.2% in high-risk patients (log rank *p* < 0.001), 5-year OS of 76% versus 40.5% (log rank *p* < 0.001), and CIR of 12.5% versus 38.4%, (*p* < 0.001).

**Figure 5:**
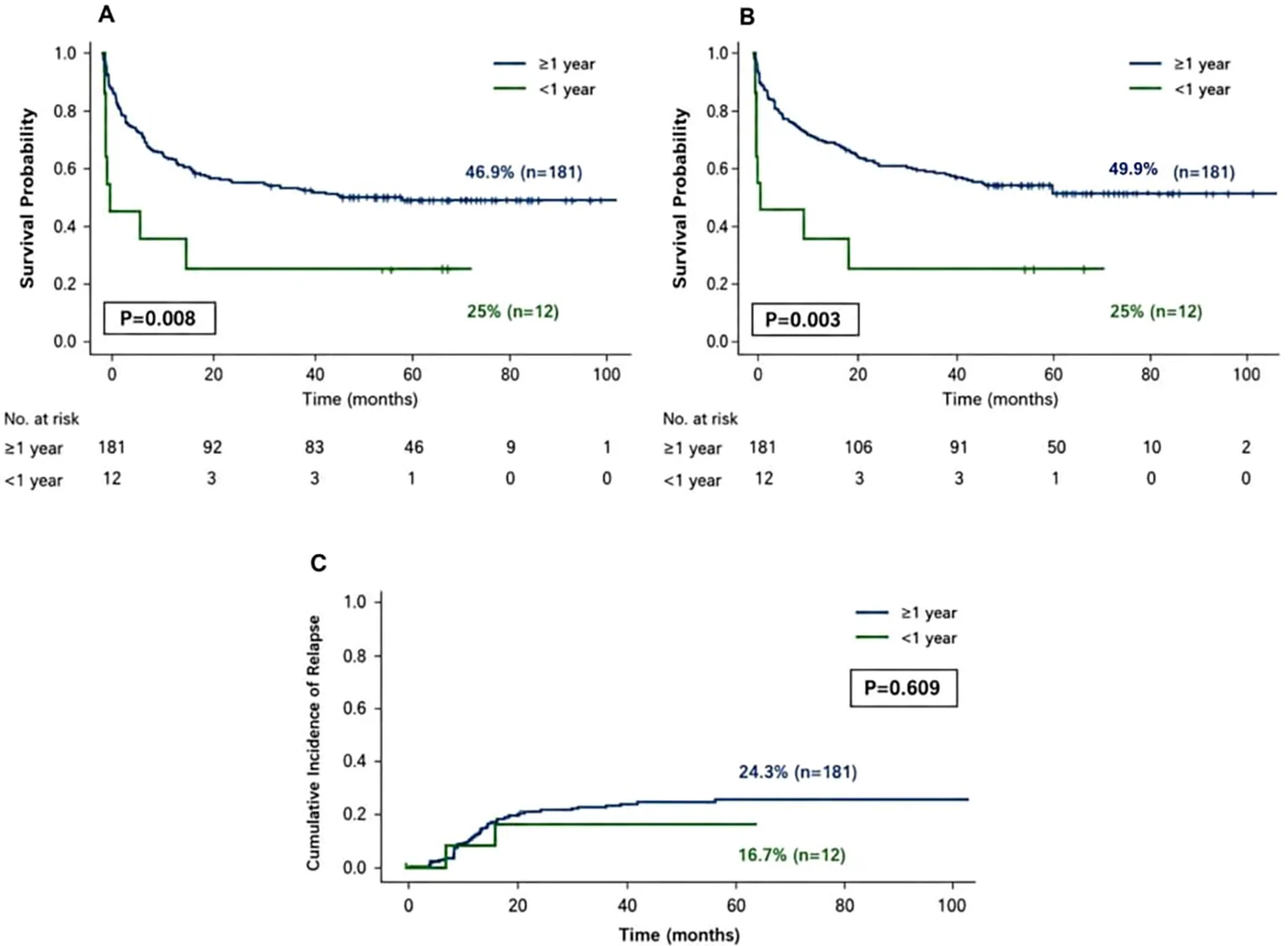
Survival and relapse according to morphologic response after Induction (I) Kaplan–Meier survival curves and cumulative incidence of relapse plots comparing patients achieving complete remission (CR, n=94) versus no CR ( n=70) post-Induction I: **(A)** Event-free survival, **(B)** overall survival, and **(C)** cumulative incidence of relapse.

**Figure 6:**
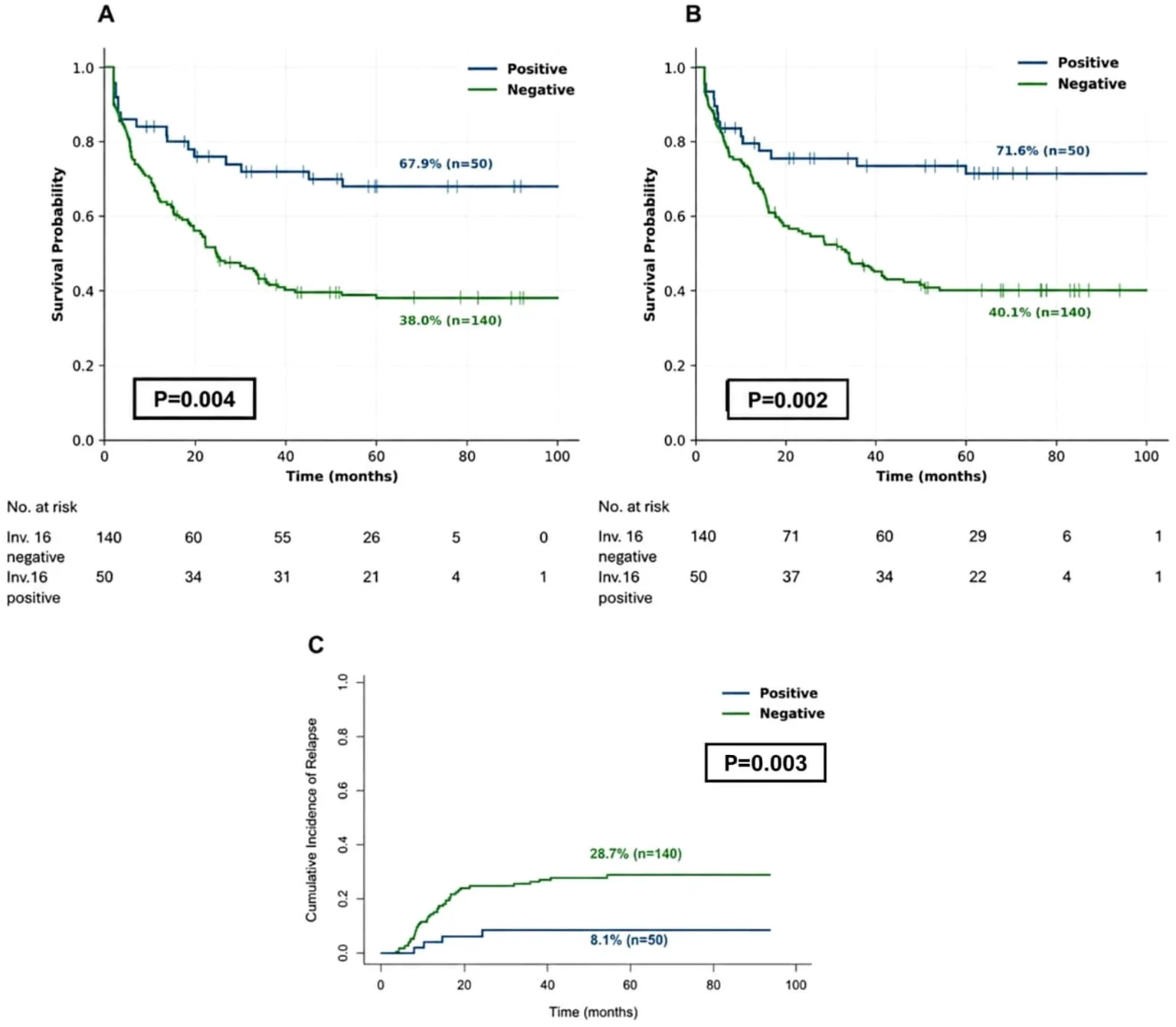
Outcomes according to flow-cytometric measurable residual disease (MRD) at End of Induction I (EOI I). Kaplan–Meier survival curves and cumulative incidence of relapse plots stratified by flow-cytometric MRD status (< 0.1% [Negative], n=26 *vs*. ≥ 0.1% [Positive], n=138) at EOI I: **(A)** 5-year EFS, **(B)** 5-year OS, and **(C)** 5-year CIR. EFS, event-free survival; OS, overall survival; CIR, cumulative incidence of relapse; CR, complete remission; MRD, measurable residual disease; EOI, end of induction.

Morphologic CR after induction II remained significantly associated with superior 5-year EFS (60.3% *vs*. 23.1%, log rank *p* < 0.001) and 5-year OS (63.0% *vs*. 38.9%, log rank *p* = 0.023), although the CIR difference was not significant (*p* = 0.680). MRD < 0.1% by flow cytometry at the end of induction II (EOI II) was associated with superior 5-year OS (67.1% *vs*. 51.6%, log rank *p* = 0.028), while 5-year EFS did not reach statistical significance (*p* = 0.128) and CIR did not differ between the 2 groups (*p* = 0.813).

Regarding induction-phase deaths (n=48, 24.9% of the cohort). Cumulative mortality, calculated from treatment initiation, was 18.8% (9/48) by day 7, 33.3% (16/48) by day 14, 52.1% (25/48) by day 21, 60.4% (29/48) by day 30, 66.7% (32/48) by day 42, and 100% (48/48) by day 70.

The 48 induction-phase deaths comprised 22/48 (45.8%) disease-related mortalities (DRM) and 26/48 (54.2%) treatment-related mortalities (TRM). Sixteen DRM occurred within 14 days of treatment initiation (early mortality), directly driven by heavy initial leukemic blast burden, tissue leukostasis, and metabolic/coagulopathic complications, hyperleukocytosis, and late presentation with delayed referral to our specialized care center. An additional 6 DRM reflected refractory disease that did not respond to treatment.

Categorized by primary organ system involvement, early disease-related mortalities ( n=16) were driven predominantly by pulmonary, metabolic, central nervous system, renal, and hemorrhagic complications. Pulmonary complications represented the leading cause (43.8%, n=7), manifesting as acute respiratory distress syndrome ( n=4), diffuse alveolar hemorrhage ( n=1), acute upper airway obstruction with stridor ( n=1), and bilateral pleural effusions with severe desaturation ( n=1). Fulminant tumor lysis syndrome accounted for 25.0% ( n=4). Central nervous system catastrophes constituted 18.8% ( n=3), comprising central nervous system hemorrhage ( n=2) and severe cerebral edema ( n=1). Massive renal blast infiltration with secondary volume overload and pulmonary edema represented 6.3% ( n=1). Lastly, coagulopathy-driven severe hemorrhage accounted for 6.3% ( n=1).

The 26 TRMs were all attributable to septic shock due to multidrug-resistant Gram-negative organisms.

Relapse occurred in 44 patients out of 139 who achieved first CR (31.7%).

### 3.5 Univariate and multivariate Cox regression analysis

In univariate analysis, age, inv(16)(p13.1;q22) or t(16;16)(p13.1;q22); CBFB::MYH11, complex karyotype, monosomy 7, hyperleukocytosis, and post-induction-I MRD were significant predictors of survival. In multivariable analysis, only inv(16)(p13.1;q22) or t(16;16)(p13.1;q22); CBFB::MYH11, monosomy 7, and MRD response at the end of induction I retained statistical significance, while age, complex karyotype, and hyperleukocytosis lost independent significance.

In univariate analysis, infant age was associated with an elevated risk of death (OS: HR 2.73, 95% CI: 1.371-5.421, *p* = 0.004) and event free survival (EFS: HR 2.48, 95% CI: 1.249-4.915, *p* = 0.009). However, in multivariate analysis, it lost significance: (OS HR 0.721, *p* = 0.650; EFS HR 0.635, *p* = 0.529) (**Table 3**).

**Table 3:** Cox proportional hazards models for overall survival and event-free survival.

Variable	OS univariable HR (95% CI)	*p*-value	OS multivariable HR (95% CI)	*p*-value	EFS univariable HR (95% CI)	*p*-value	EFS multivariable HR (95% CI)	*p*-value
Age, <1 year *vs*. ≥1 year	2.73 (1.371-5.421)	0.004*	0.721 (0.175–2.967)	0.650	2.48 (1.249-4.915)	0.009*	0.635 (0.155–2.611)	0.529
inv(16)(p13.1;q22) or t(16;16)(p13.1;q22); CBFB::MYH11 positive *vs*. negative	0.42 (0.238–0.742)	0.003*	0.253 (0.108–0.592)	0.002*	0.456 (0.267–0.779)	0.004*	0.309 (0.146–0.653)	0.002*
Complex karyotype, positive *vs*. negative	2.845 (1.042–7.765)	0.041*	2.163 (0.522–8.962)	0.287	2.579 (0.947–7.023)	0.064	1.928 (0.467–7.968)	0.364
Monosomy 7, positive *vs*. negative	3.518 (1.692–7.313)	<0.001*	6.206 (2.692–14.306)	<0.001*	3.432 (1.655–7.117)	<0.001*	5.789 (2.534–13.227)	<0.001*
Hyperleukocytosis (WBC ≥100 × 10^9^/L), yes *vs*. no	1.63 (1.039–2.549)	0.034*	1.547 (0.846–2.829)	0.156	1.59 (1.024–2.464)	0.039*	1.503 (0.840–2.689)	0.170
MRD after Induction I positive *vs*. negative	2.838 (1.141–7.059)	0.025*	2.868 (1.133–7.260)	0.026*	2.208 (1.014–4.810)	0.046*	2.289 (1.030–5.083)	0.042*

HR, hazard ratio; CI, confidence interval; OS, overall survival; EFS, event-free survival; WBC, white blood cell count. Multivariable Cox proportional hazards regression used the Efron method. Hyperleukocytosis was defined as WBC ≥100 × 10^9^/L. Age was dichotomized as <1 year vs. ≥1 year for the univariate and multivariable Cox models, based on the established prognostic significance of infant age at diagnosis. *significant p value <0.05.

Patients with inv(16)(p13.1;q22) or t(16;16)(p13.1;q22); CBFB::MYH11 were associated with significantly improved OS (HR 0.42, 95% CI: 0.238-0.742, *p* = 0.003) and EFS (HR 0.456, 95% CI: 0.267-0.779, *p* = 0.004) on univariate analysis. This association was retained and strengthened on the multivariable analysis (OS HR 0.253, 95% CI 0.108-0.592, *p* = 0.002; EFS HR 0.309, 95% CI: 0.146-0.653, *p* = 0.002), confirming inv(16)(p13.1;q22) or t(16;16)(p13.1;q22); CBFB::MYH11 as an independent favorable prognostic factor.

Monosomy 7 was demonstrated as a consistent adverse prognostic factor. Univariable HRs for OS and EFS were 3.518 (95% CI: 1.692-7.313, *p* < 0.001) and 3.432 95% CI: 1.655-7.117, *p* < 0.001), respectively, and remained significant in multivariable analysis (OS HR 6.206, 95% CI 2.692-14.306, *p* < 0.001; EFS HR 5.789, 95% CI: 2.534-13.227, *p* < 0.001).

MRD positivity after induction I was significantly associated with inferior OS and EFS on both univariable (OS HR 2.838, 95% CI 1.141-7.059, *p* = 0.025; EFS HR 2.208, 95% CI: 1.014-4.810, *p* = 0.046), and multivariable analysis (OS HR 2.868, 95% CI 1.133-7.260, *p* = 0.026; EFS HR 2.289, 95% CI: 1.030-5.083, *p* = 0.042), confirming MRD EOI I as an independent prognostic factor.

Complex karyotype ( n=5) was associated with inferior overall survival (OS) on univariable analysis ( p=0.041), though it did not retain statistical significance on multivariable analysis. Among these five cases, two patients died during first induction, one experienced refractory disease, one relapsed, and one remained in continuous complete remission at last follow-up. *KMT2A* rearrangement demonstrated decreased survival that did not reach statistical significance. Similarly, *FLT3*-ITD ( n=8) showed decreased survival that did not reach statistical significance. Monosomy 5 ( n=1) and *TP53* mutations ( n=3) were identified in very few patients. Notably, *TP53* mutations were associated with a pronounced poor outcome: two patients had refractory disease and one relapsed, with all three dying within 3 years of follow-up. Given the small number of positive cases for these high-risk alterations, which reflects the distinct molecular landscape of pediatric monocytic AML, survival associations for these specific subgroups should be interpreted with caution.

Complete 5-year event-free survival, overall survival, and cumulative incidence of relapse estimates across all clinical, cytogenetic, and treatment response variables are detailed in **Supplementary Table 1**.

We analyzed the association of early deaths with age at diagnosis, and presence of hyperleukocytosis: early death (≤14 days from treatment initiation, n=16) was strongly associated with infancy (50.0% vs. 5.5% in older children; OR 16.50, 95% CI 3.71–75.24, P < 0.001), and hyperleukocytosis (23.8% vs. 4.0%; OR 7.44, 95% CI 2.26–26.85, P < 0.001).

## 4 Discussion

In this retrospective cohort study of 193 pediatric patients with acute myeloid leukemia (AML) of monocytic differentiation (FAB M4 and M5 subtypes), we identified several key prognostic factors and survival trends. This represents one of the largest single-center analyses of monocytic AML subtypes in a resource-limited setting.

Age distribution was bimodal, peaking in early childhood and adolescence, 59.1% fell within 1–9 years, and infants (< 1 year) comprised 6.2%. Puumala et al. reported a bimodal age distribution, with higher incidence in patients younger than 2 years and a second peak in 10 to 20 years old (28). M5 was more common in infants (14.5% *vs*. 2.3% in M4), consistent with Masetti et al., who reported infant M5 predominance (20/63 *vs*. 10/63 M4) (29).

Infants had significantly poorer survival and significantly lower 5-year OS and EFS survival, and insignificant CIR as their inferior 5-year OS and EFS, was driven instead by higher non-relapse mortality, consistent with known infant vulnerability to treatment-related toxicity (30). Wang et al. reported that infants have the poorest survival in AML (31), while Blaise et al. reported a poorer survival in infants < 2 years, though not reaching statistical significance (32).

Hyperleukocytosis significantly affected 5-year OS and EFS. Additionally, early disease related mortalities were linked to hyperleukocytosis at diagnosis. Pawińska-Wąsikowska et al. described hyperleukocytosis as the main cause of early mortality, and reported leukostasis and bleeding as the first cause of mortality in a cohort of 646 of children with AML (33). Zeller et al. described the role of leukostatis in infarction, pulmonary involvement and bleeding (34). Additionally, Xu et al. reported significantly inferior 5-year event-free survival (EFS) (P < 0.001) and a trend of inferior 5-year overall survival (OS) (P = 0.059) in children presented with hyperleukocytosis (35).

Complex karyotype (2.6%, 5/190) was less frequent than reported by Chen et al. and Fleishman et al. (12–13.8%) (36, 37). and was significantly associated with inferior OS, consistent with Chen et al. and Grimwade et al. (36, 38). although the small subgrou*p* warrants cautious interpretation. Monosomy 7 occurred in 8 cases (4.2%) consistent with its reported rarity in pediatric AML (39, 40). Its adverse impact on survival is well documented (7, 40). Quessada et al. reported monosomy 7 adverse outcome to be mainly attributed to resistance to induction therapy (41). In our cohort, 100% of monosomy 7 cases experienced events, of them 2 had a refractory disease, 5 died during treatment and one relapsed, with no survivors beyond 42.8 months of diagnosis. Monosomy 5 (1 patient, died early) was rarer than the 1.2% reported by Johnston et al., whose larger cohort linked it to inferior OS/EFS (42). TP53 mutation (3 patients, all died within 3 years) confirmed its established adverse role (43, 44).

KMT2A-rearrangement occurred in 25.8%, within the reported range of (21-30%) in pediatric AML (12, 45). It was significantly more in infants less than 1 year (63.6 *vs*. 23.5, *p* = 0.003), in line with Zwaan et al. and Mendoza-Castrejon et al., who reported its enrichment in infants (12, 46). Patients had lower EFS and OS, not reaching statistical significance, in line with KMT2A heterogenicity and variable outcomes depending on fusion partner (47). FLT3-ITD similarly trended toward worse OS/EFS without reaching significance, mostly due to our small sample *(n* = 8), and in line with its established adverse role on survival (48, 49).

MRD positivity after induction I predicted worse 5-year OS (52.3% *vs*. 80.4%) and EFS (50.0% *vs*. 72.1%), and borderline significance for CIR (*p* = 0.050). It was also independently prognostic on multivariable analysis, matching Rubnitz et al., Brodersen et al., and San Miguel et al., who each identified early MRD as among the strongest independent prognostic factors in childhood AML (26, 50, 51).

inv(16)(p13.1;q22) or t(16;16)(p13.1;q22); CBFB::MYH11 (50/190 = 26.3%) was significantly more common in M4 (34.1%) than M5 (9.8%), and far exceeding the 7% reported by Harrison et al. and Grimwade et al. (7, 38), likely reflecting our monocytic-subtype focus, and consistent with Qiu et al.’s finding that > 98% of inv(16) cases occur in M4 (52). inv(16)(p13.1;q22) or t(16;16)(p13.1;q22); CBFB::MYH11- positive patients had significantly better survival on univariable and multivariable analysis, in line with published studies (5, 52). t(8;21) (q22;q22.1); RUNX1::RUNX1T1(10.2% of M4, absent in M5) matched its known M2 association and monocytic-subtype rarity (53), EFS was significantly superior (p=0.048), though OS was not, reinforcing its reported favorable role (52, 54). NPM1 mutations trended toward better OS/EFS without significance (*n* = 7), likely reflecting sample size, consistent with mixed prior findings of Hollink et al. reporting favorable outcome for EFS, not OS; and Srinivasan et al. reporting favorable overall survival with NPM1 (55, 56). All 3 CEBPA-positive patients remained alive and event-free, matching reports linking CEBPA (especially biallelic) mutations to favorable outcomes (57, 58). WT1 overexpression was significantly more common in M4, but showed no significant survival impact, consistent with Ho et al. and Noronha et al. reports (59, 60).

Overall survival in our cohort (5-year OS: 48.3%) was lower than the 75% 5-year OS reported in high-income settings (61, 62). However, it was consistent with Van Weelderen et al.’s systematic review of LMIC outcomes, reporting that a lack of essential resources for supportive care as an important cause that these improved survival levels are not met in LMICs (18). Nyeko et al. reported 16.7% 5-year OS in a retrospective study on 159 pediatric AML cases in Uganda (17). Mamo et al. reported a 23% 3-year OS in a cohort of 92 AML children in Ethiopia (20). 5-year EFS in whole cohort (45.5%), 39.2% in high risk, was lower than the significantly improved EFS rates (>50%) in COG trials in high risk groups (45, 63), but within reported rates in LMICs (24% to 63%) (64–66). M4 patients showed numerically superior 5-year EFS (48.2% *vs*. 40.1%) and OS (51.9% *vs*. 40.6%) compared with M5, though neither difference reached statistical significance (*p* = 0.195 and *p* = 0.086, respectively), reinforcing the importance of a cytogenetic and response based risk stratification (3, 36, 38). Induction mortality (24.9%) exceeded high-income countries reported rates of (5–15%) (33). but remained below LMIC (29.3–30.3%) (18–20). Crude relapse rate (31.7%) was within reported range of (24 - 41%) (3, 4). Post-induction-II CR (91.4%, 139/152) exceeded the LMIC median of 55.0%, and reported rates in Africa (23.1 - 88.9%) in a literature research that included 2230 children with AML (19). It closely matched rates in HICs (85-95%), mostly reflecting the role of supportive care (67, 68). HSCT outcomes (11/14 in continuous CR) support its curative potential in selected high-risk patients, consistent with findings of Algeri et al., Bunin et al., Pession et al. Sarper et al. (69–72).

The early mortality observed during induction in our cohort highlights a recognized challenge with the aggressiveness of this subtype of childhood acute leukemia as described by Varotto et al. (13) and Cencelewicz et al. (74), and resonates with broader literature findings in regional pediatric oncology centers on pediatric acute leukemias in developing health systems, such as Solmaz et al. (73), which emphasizes that survival in regional treatment units is heavily dictated by baseline clinical presentation, high-risk stratification, and local supportive care infrastructure, where patients with acute leukemias frequently present with advanced disease burden, strengthening upfront supportive care algorithms, remains essential to reducing induction deaths (13, 73, 74).

In our cohort, all induction phase treatment-related mortalities were driven by multidrug-resistant Gram-negative septic shock. This aligns directly with previously published data from our center (Mbatia et al., (75)), which reported a high prevalence of multidrug-resistant Gram-negative infections accounting for 80% of patients with documented bloodstream infections among pediatric patients with AML.

The limitations of our research arise from its retrospective design. The analysis was constrained by a small sample size and fragmented subgroups. Findings require validation in a larger multicenter cohort, especially for rare but high-risk genomic subgroups. As next-generation sequencing and molecular MRD approaches continue to expand, future research is expected to improve risk stratification, enhance accurate MRD detection, and identify previously undetected (cryptic) fusion genes and gene mutations that may further influence outcomes. In conclusion, this study demonstrates that outcomes in pediatric monocytic AML are driven primarily by cytogenetic and molecular features and by early therapeutic response. inv(16)(p13.1;q22) or t(16;16)(p13.1;q22); CBFB::MYH11, monosomy 7, and MRD positivity post-induction I are the only independent predictors of overall and event-free survival. Another limitation of our study is the inability to formally assess the impact of nutritional status on induction mortality, as these data were not uniformly documented across the cohort. In addition, the relatively small number of patients who underwent HSCT ( n=14/99, 14.1% of high-risk cases) was limited by the availability of matched sibling donors. Consequently, our findings regarding HSCT efficacy represent observational trends rather than definitive evidence of therapeutic benefit, highlighting the need for further prospective evaluation in larger, multi-center cohorts. Finally, while all patients received a uniform therapeutic protocol and standardized supportive management, this 6-year study timeframe naturally encompassed incremental evolutions in diagnostic sensitivity, minimal residual disease (MRD) monitoring, and general clinical care. Additionally, because supportive care regimens, transplantation strategies, and infant-specific reduced-toxicity protocols were not evaluated as pre-specified primary endpoints, findings regarding these modalities remain observational and require cautious interpretation.

## Data Availability

The raw data supporting the conclusions of this article will be made available by the authors, without undue reservation.
