# Supplementary material for: Clinical outcomes and prognostic factors in pediatric acute myeloid leukemia with monocytic differentiation: a single-center cohort study from Egypt
Source: Front Oncol. 2026 Oct 12;16:1969723. doi: 10.3389/fonc.2026.1969723 (PMC13696307; doi:10.3389/fonc.2026.1969723)
Supplement: Supplementary file 1 [file DataSheet1.docx]

Supplementary material

**Title:** Clinical Outcomes and Prognostic Factors in Pediatric Acute Myeloid Leukemia with Monocytic Differentiation: A Single-Center Cohort Study from Egypt

**Authors:** Tarek Ahmed^1*^, Mahmoud Hammad^2,3*^, Sonya Soliman^4,5^, Nahla Elsharkawy^4,5^, Sherine Ibrahim Salem^4,5^, Sonia Ahmed^2,3^**^†^** and Alaa El-Haddad^2,3^**^†^**

1. Department of Clinical Oncology and Nuclear Medicine, Sohag University, Sohag, Egypt.

2. Department of Pediatric Oncology, National Cancer Institute (NCI), Cairo University, Cairo, Egypt.

3. Department of Pediatric Oncology, Children’s Cancer Hospital, Egypt-57357, Cairo, Egypt.

4. Department of Clinical Pathology, National Cancer Institute, Cairo University, Cairo, Egypt.

5. Department of Clinical Pathology, Children’s Cancer Hospital, Egypt-57357, Cairo, Egypt.

**Both authors contributed equally to this work as the first author.*

**^†^***These authors have contributed equally to this work and share last authorship*

**Corresponding author: Tarek Ahmed**

Email: tarek.ahmed@med.sohag.edu.eg

Tel: +201098984549

**Co-corresponding author: Sonia Ahmed**

Email: sonia.ahmed@nci.cu.edu.eg

Tel: +201222248495

**Supplementary Figure 1: CCHE-57357 pediatric acute myeloid leukemia (AML) treatment roadmap and risk stratification strategy.**

**Legend:**

Flowchart detailing the CCHE-57357 treatment protocol adapted from the Children’s Oncology Group (COG) AAML1031 protocol framework. All patients receive initial diagnostic cytogenetic/molecular workup and Induction I ($A_{10}D_{3}E_{5}$ + triple intrathecal therapy [ITH]). Risk stratification into Low Risk (LR) or High Risk (HR) is determined by initial genetics/cytogenetics and flow-cytometric measurable residual disease (MRD) status at the end of Induction I (EOI I threshold: 0.1%).

Induction II is stratified by risk ($A_{8}D_{3}E_{5}$ + ITH for LR; MA + ITH for HR). Following remission assessment, all patients in complete remission (CR) proceed to Intensification I (AE + ITH). Final consolidation (Intensification II) assigns HR patients with a matched donor to hematopoietic stem cell transplantation (SCT), HR patients without a donor to HD-ARAC/L-ASP, and LR patients to MA + ITH.

**Abbreviations & Regimen Definitions:**

- $A_{10}D_{3}E_{5}$ **/** $A_{8}D_{3}E_{5}$**:** Cytarabine (Ara-C; 10 or 8 days), Idarubicin (3 days), Etoposide (5 days).
- **MA:** High-dose Cytarabine (4 days) + Mitoxantrone (3 days).
- **AE:** High-dose Cytarabine (5 days) + Etoposide (5 days).
- **HD-ARAC / L-ASP:** High-dose Cytarabine + L-Asparaginase.
- **ITH:** Triple Intrathecal Therapy (Methotrexate, Cytarabine, Hydrocortisone).

**AML:** Acute Myeloid Leukemia; **BMA:** Bone Marrow Aspirate; **CCHE:** Children’s Cancer Hospital Egypt; **CR:** Complete Remission; **MRD:** Measurable Residual Disease; **SCT:** Stem Cell Transplantation.

Supplementary Table 1: Five-year EFS, OS, and cumulative incidence of relapse

| **Characteristic** | **5-year EFS** **% (95% CI)** | ***p*-value** | **5-year OS** **% (95% CI)** | ***p*-value** | **5-year CIR** **% (95% CI)** | ***p*-value** |
| --- | --- | --- | --- | --- | --- | --- |
| Whole cohort | 45.5% (39.0–53.0) |  | 48.3% (42.0–56.0) |  | 23.8% (17.7–30.2) |  |
| **Age, years** | | | | | | |
| <1 | 25.0% (9.4–67.0) | 0.028* | 25.0% (9.0–67.0) | 0.012* | 16.7% (0.0-40.3) | 0.321* |
| 1 to <10 | 46.6% (38.0–57.0) |  | 49.8% (41.0–60.0) |  | 28.0% (19.5-36.5) |  |
| >10 | 47.5% (37.0–61.0) |  | 49.8% (39.0–63.0) |  | 18.0% (8.7-27.4) |  |
| <1 year vs. ≥1year | 25.0% vs. 46.9% (9.4-66.6) vs. (40.1-54.9) | 0.008* | 25% vs. 49.9% (9.4-66.6) vs. (42.9-57.9) | 0.003* | 16.7% vs. 23.8% (0.0-40.3 vs. 17.4-30.1) | 0.637 |
| **Sex** | | | | | | |
| Male | 50.2% (41.0–61.0) | 0.191 | 50.6% (42.0–62.0) | 0.310 | 22.5% (14.3-30.7) | 0.774 |
| Female | 40.0% (31.0–52.0) |  | 45.6% (36.0–58.0) |  | 25.6% (16.1- 35.0) |  |
| **WBC count, × 10⁹/L** |  | | | | | |
| <100 | 48.5% (41.0–57.0) | 0.037* | 51.7% (44.0–60.0) | 0.032* | 23.0% (16.1-29.9) | 0.492 |
| ≥100 | 35.1% (23.0–53.0) |  | 35.8% (23.0–55.0) |  | 26.8% (12.9-40.7) |  |
| **FAB classification** |  | | | | | |
| M4 | 48.2% (40.0–57.0) | 0.195 | 51.9% (44.0–61.0) | 0.086 | 24.2% (16.7-31.8) | 0.859 |
| M5 | 40.1% (29.0–54.0) |  | 40.6% (30.0–56.0) |  | 22.8% (12.1-33.5) |  |
| **Cytogenetic and molecular findings** |  | | | | | |
| **Favorable** |  |  |  |  |  |  |
| inv(16) or t(16;16) | 67.9% (56.0–82.0) | 0.004* | 71.6% (60.0–86.0) | 0.002* | 8.1% (0.4-15.7) | 0.003* |
| t(8;21) | 76.9% (57.0–100) | 0.048* | 76.9% (57.0–100) | 0.071 | 7.7%  (0.0–22.9) | 0.186 |
| NPM1 mutation | 71.4% (45.0–100) | 0.225 | 71.4% (45.0–100) | 0.310 | NE | NE |
| CEBPA mutation | 100% (100–100) | NE | 100% (100–100) | NE | NE | NE |
| **Intermediate** |  | | | | | |
| Normal karyotype | 46.5% (33.0–65.0) | 0.750 | 47.8% (35.0–66.0) | 0.830 | 29.1% (14.6–43.5) | 0.349 |
| KMT2A rearrangement | 38.0% (27.0–55.0) | 0.106 | 39.0% (27.0–56.0) | 0.070 | 27.3% (14.4–40.2) | 0.381 |
| **Unfavorable** |  | | | | | |
| Monosomy 7 | 0%  *(No patients survived beyond 42.8 months)* | <0.001* | 0% *(No patients survived beyond 42.8 months)* | <0.001* | 12.5%  (0.0–66.8%) | 0.568 |
| Monosomy 5 | 0% *(one early death)* | NE | 0% *(one early death)* | NE | NE | NE |
| Complex karyotype | 20.0% (3.5–100) | 0.055 | 20.0% (3.5–100) | 0.033* | 20.0%  (0.0–66.8) | 0.918 |
| FLT3-ITD | 28.6% (8.9–92.2) | 0.860 | 25.0% (7.5–83.0) | 0.350 | 62.5%  (23.9–100.0) | 0.012†* |
| TP53 mutation | 0% *(all 3 cases died within 3 years of diagnosis)* | NE | 0% *(all 3 cases died within 3 years of diagnosis)* | NE | NE | NE |
| **WT1 overexpression** |  | | | | | |
| Yes | 49.8% (39.5–63.0) | 0.360 | 52.4% (42.0–66.0) | 0.227 | 21.0%  (11.4–30.6) | 0.817 |
| No | 38.1% (22.1–66.0) |  | 38.1% (22.0–66.0) |  | 23.8%  (4.7–42.9) |  |
| **Morphologic response after induction I** |  | | | | | |
| Complete remission (CR) | 62.4% (53.0–73.0) | 0.013* | 64.8% (56.0–76.0) | 0.023* | 20.5%  (12.2–28.8) | 0.025* |
| No CR | 41.3% (31.0–56.0) |  | 46.6% (36.0–61.0) |  | 37.3%  (25.2–49.4) |  |
| **MRD by flow cytometry at EOI I** |  | | | | | |
| <0.1% | 72.1% (56.5–92.1) | 0.041* | 80.4% (66.3–97.4) | 0.019* | 12.1%  (0.0–25.3) | 0.050* |
| ≥0.1% | 50% (42.2–59.3) |  | 52.3% (44.3–61.7) |  | 30.4%  (23.2–39.1) |  |
| **Risk stratification at EOI I** |  | | | | | |
| Low | 70.4% (60.0–82.0) | <0.001* | 76.1% (67.0–87.0) | <0.001* | 12.5%  (4.3–20.6) | <0.001* |
| High | 39.2% (30.0–50.0) |  | 40.5% (31.0–52.0) |  | 38.4%  (28.5–48.4) |  |
| **Morphologic response after induction II** |  | | | | | |
| Complete remission (CR) | 60.3% (53.0–69.0) | <0.001* | 63.0% (55.0–72.0) | 0.013* | 31.0% (23.1–38.9) | 0.680 |
| No CR | 23.1% (8.0–62.0) |  | 38.9% (20.0–74.0) |  | 23.1% (0.0–48.3) |  |
| **MRD by flow cytometry at EOI II** |  | | | | | |
| <0.1% | 61.0% (51.4–72.3) | 0.128 | 67.1% (57.7–78.1) | 0.028* | 31.1% (21.2–41.0) | 0.813 |
| ≥0.1% | 51.6% (40.6–65.7) |  | 51.6% (40.6–65.7) |  | 29.0% (17.6–40.5) |  |

**Note.** EFS, event-free survival; OS, overall survival; CIR, cumulative incidence of relapse; MRD, measurable residual disease; EOI, end of induction; NE, not evaluable (subgroup too small ($n<5$) for reliable statistical comparison). A 0% EFS or OS estimate indicates that all observed patients experienced the relevant event prior to 5 years. *p* values were calculated using the log-rank test for survival outcomes (EFS, OS) and Gray's test for CIR. Wide confidence intervals reflect the limited number of patients remaining at risk at this time point. Estimates for complex karyotype should be interpreted with caution due to the small sample size ($n=5$). In the MRD $\geq0.1\%$ subgroup at EOI II, the 5-year EFS and OS point estimates are numerically identical (51.6%, 95% CI: 40.6–65.7); this reflects two distinct event patterns (differing timing and distribution of relapse versus death events over follow-up) rather than a duplicated calculation, as confirmed by separate Kaplan–Meier life tables for each endpoint.

${}^{\dagger}{}$ Significant *p* value to be interpreted with caution due to the small sample size ($n=8$).
*significant *p* value < 0.05
